# SpikeAtConv: an integrated spiking-convolutional attention architecture for energy-efficient neuromorphic vision processing

**DOI:** 10.3389/fnins.2025.1536771

**Published:** 2025-03-12

**Authors:** Wangdan Liao, Fei Chen, Changyue Liu, Weidong Wang, Hongyun Liu

**Affiliations:** ^1^School of Biological Science and Medical Engineering, Beihang University, Beijing, China; ^2^Medical Innovation Research Division, Chinese PLA General Hospital, Beijing, China; ^3^Key Laboratory of Biomedical Engineering and Translational Medicine, Chinese PLA General Hospital, Beijing, China

**Keywords:** spiking neural network, self-attention, convolutional neural network, deep learning, computer vision

## Abstract

**Introduction:**

Spiking Neural Networks (SNNs) offer a biologically inspired alternative to conventional artificial neural networks, with potential advantages in power efficiency due to their event-driven computation. Despite their promise, SNNs have yet to achieve competitive performance on complex visual tasks, such as image classification.

**Methods:**

This study introduces a novel SNN architecture called SpikeAtConv, designed to enhance computational efficacy and task accuracy. The architecture features optimized spiking modules that facilitate the processing of spatio-temporal patterns in visual data, aiming to reconcile the computational demands of high-level vision tasks with the energy-efficient processing of SNNs.

**Results:**

Extensive experiments show that the proposed SpikeAtConv architecture outperforms or is comparable to the state-of-the-art SNNs on the datasets. Notably, we achieved a top-1 accuracy of 81.23% on ImageNet-1K using the directly trained Large SpikeAtConv, which is a state-of-the-art result in the field of SNN.

**Discussion:**

Our evaluations on standard image classification benchmarks indicate that the proposed architecture narrows the performance gap with traditional neural networks, providing insights into the design of more efficient and capable neuromorphic computing systems.

## 1 Introduction

Spiking Neural Networks (SNNs) represent the forefront of a paradigm shift toward more energy-efficient and biologically plausible computational models. As the third generation of neural network technologies, SNNs offer a promising alternative to traditional machine intelligence systems by emulating the event-driven characteristics of biological neural processing (Maass, 1997). The appeal of SNNs is multifaceted, with their ability not only to operate at lower power consumption, but also to perform computations in a manner that closely mirrors the spatiotemporal dynamics of the brain (Roy et al., 2019). The spike-based communication protocol of SNNs is especially well-suited for sparse and asynchronous computations, making it highly appropriate for deployment on neuromorphic chips. These chips are designed to emulate the neural architecture of the brain, leveraging the inherent sparse activation patterns of SNNs to achieve significant energy efficiency improvements (Li et al., 2024; Frenkel et al., 2023; Merolla et al., 2014; Davies et al., 2018; Pei et al., 2019).

Despite their potential, SNNs have historically grappled with performance limitations, particularly in complex cognitive tasks that are easily handled by their Artificial Neural Networks (ANNs) counterparts. This has prompted researchers to explore the adaptation of successful ANN architectures into the spiking domain. For instance, SNNs based on Convolutional Neural Networks (CNNs) have been developed, enabling the transposition of classic architectures like VGG and ResNet into SNN frameworks (LeCun et al., 1989; Wu et al., 2021). These adaptations have made significant strides, yet the quest for architectures that can fully exploit the unique advantages of SNNs continues.

The emergence of the Transformer architecture, originally designed for natural language processing, has sparked a new wave of innovations across various fields of machine learning (Vaswani et al., 2017). Its success in ANNs has not gone unnoticed in the SNNs community, leading to the exploration of Transformer-based designs within spiking networks (Zhang et al., 2022; Zhou et al., 2023). However, the integration of the self-attention mechanism into SNNs has been challenging, as it relies on operations that are at odds with the principles of spike-based processing, such as the energy-intensive Multiply-and-Accumulate (MAC) operations. Recent efforts have sought to reconcile this discrepancy by proposing spike-driven variants of the self-attention mechanism, aiming to retain the computational efficiency and low power consumption that are hallmarks of SNNs (Yao et al., 2023). These innovations represent a significant departure from traditional Transformer models, yet the challenge remains to demonstrate their superiority over existing SNN designs in both performance and energy efficiency.

**Figure 1:**
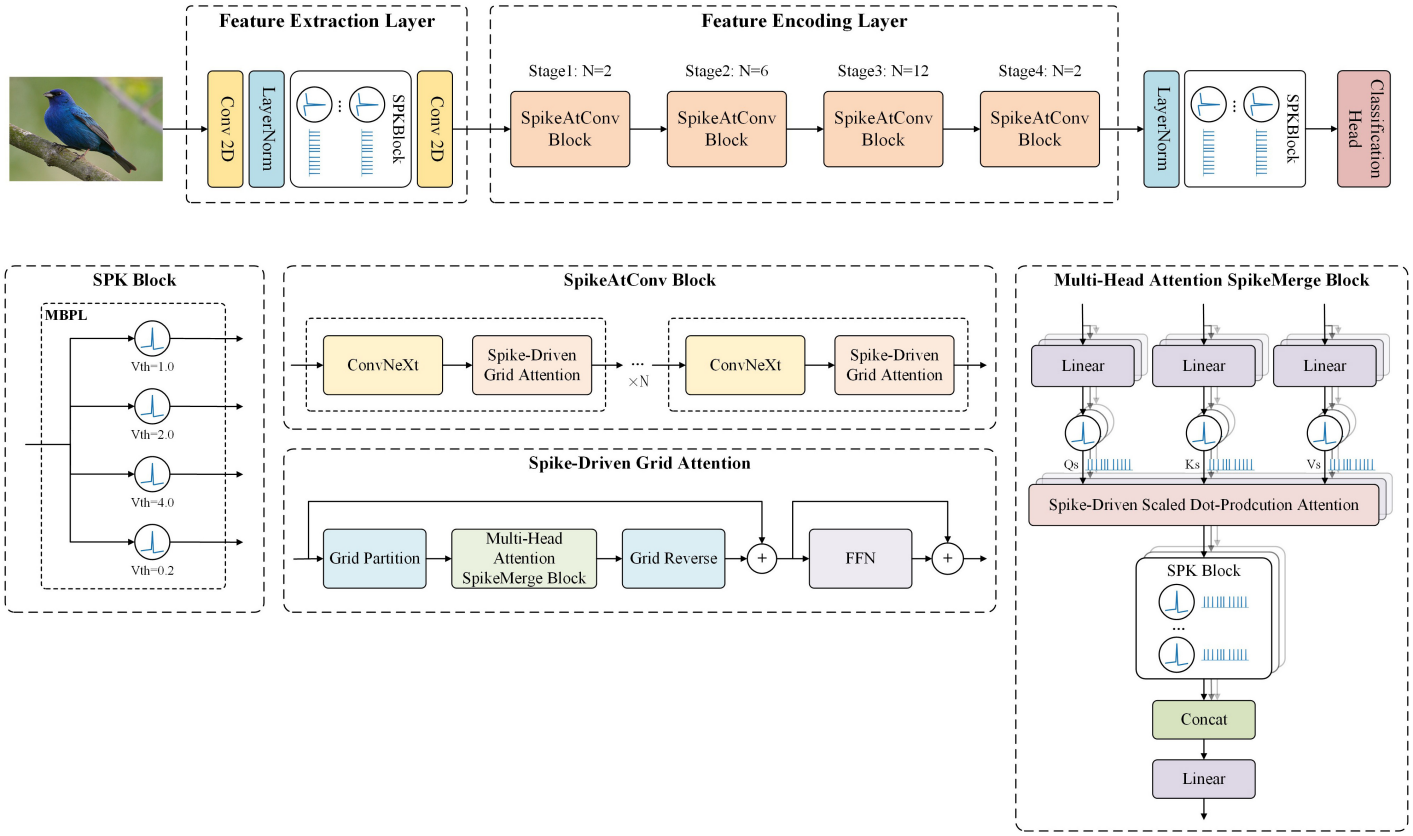
The overview of SpikeAtConv. The framework is primarily composed of three components: the Feature Extraction Layer, the Feature Encoding Layer, and the Decision Layer. Initially, the input image is subjected to preliminary processing within the Feature Extraction Layer, where essential characteristics are identified. Subsequently, the Feature Encoding Layer performs a comprehensive analysis to distill salient features from the extracted data. Finally, the decision layer synthesizes this information to generate the prediction results.

In this paper, we introduce an innovative spiking neural network framework called SpikeAtConv, designed to incorporate the strengths of advanced Transformer models into SNNs. An overview of the SpikeAtConv network is shown in Figure 1. Inspired by MaxViT, we propose a novel spike-driven transformer module named Spike-Driven Grid Attention. This module facilitates global spatial interactions within a single block, providing enhanced flexibility and efficiency compared to traditional spike-driven full self-attention or (shifted) window/local attention mechanisms. The SpikeAtConv Block, composed of Spike-Driven Grid Attention and ConvNeXt, serves as the core component of the SpikeAtConv network. Additionally, we have designed various Spiking Neuron (SPK) Blocks to enable a more flexible neuron activation mechanism, such as the Multi-Branch Parallel LIF SPK (MBPL) Block, which consists of multiple parallel neurons with different thresholds.

The main contributions of this paper are as follows:

1. We design a series of SPK Blocks to explore the effects of multiple neurons with different thresholds and combinations on network performance. Through extensive experiments, we identify the optimal configuration of the SPK module, which significantly enhances the computational performance of the model.
2. We develop Spike-Driven Grid Attention, enabling global spatial interactions within a single block. This allows the SpikeAtConv block to capture both local and global significant features more effectively.
3. We propose the SpikeAtConv network, which is based on the developed SPK Block and SpikeAtConv Block. This architecture successfully adapts advanced transformer models to the SNN framework, thereby enhancing the computational performance and efficiency of the model.
4. Extensive eperiments show that the proposed model outperforms or is comparable to the state-of-the-art (SOTA) SNNs on the datasets. Notably, we achieved a top-1 accuracy of 81.23% on ImageNet-1K using the directly trained Large SpikeAtConv, which is a SOTA result in the field of SNN.

## 2 Related work

SNNs have emerged as a promising alternative to traditional ANNs due to their potential for energy-efficient and biologically plausible computations. This section reviews key advancements in ANN-to-SNN conversion techniques, direct training of SNNs, and the integration of Vision Transformer (ViT) within SNN frameworks.

### 2.1 ANN-to-SNN conversion

ANN-to-SNN conversion techniques leverage established training methodologies of ANNs to initialize SNNs. These methods aim to translate trained ANN weights into their SNN counterparts, thereby inheriting the performance characteristics of ANNs while benefiting from the energy efficiency of SNNs. Pioneering work by Deng and Gu (2021) replaced ReLU activation functions with Integrate-and-Fire (IF) neurons to facilitate the conversion process. Subsequent enhancements by Hu et al. (2024) introduced weight scaling and normalization, effectively narrowing the performance gap between ANNs and SNNs.

Further advancements by Rueckauer et al. (2017) and Han et al. (2020) incorporated soft reset mechanisms, preserving temporal information and minimizing spike count errors associated with hard reset strategies. Additionally, dynamic threshold adjustment strategies proposed by Sengupta et al. (2019) and Zhang et al. (2023) enhanced the adaptability of SNNs to varying activation regimes during conversion. Bu et al. (2022) demonstrated that initializing membrane potentials at half the threshold could significantly reduce conversion inaccuracies.

Despite the success of ANN-to-SNN conversion methods in replicating ANN performance, they remain inherently limited by the constraints of the source ANNs, such as dependency on specific architectures and the inability to fully exploit the temporal dynamics intrinsic to SNNs. Therefore, while effective for performance replication, these methods may not fully harness the unique advantages of SNNs in temporal processing and energy efficiency.

### 2.2 Direct training of SNNs

Direct training of SNNs enables end-to-end optimization, circumventing the limitations of conversion techniques. Various strategies have been developed to address the non-differentiability of spike activations, including quantization and binarization approaches (Li et al., 2019, 2021a), adder neural networks (Chen et al., 2020), and probabilistic models (Amir et al., 2017; Bengio et al., 2013). Rate-encoding networks quantify spike rates for gradient calculation (Lee et al., 2016; Neftci et al., 2017). Surrogate gradient tries to find an alternative differentiable surrogate function to replace the undifferentiable firing activity when doing back-propagation of the spiking neurons (Guo et al., 2023).

Surrogate gradient methods have proven particularly effective, with Wu et al. (2018) and Neftci et al. (2019) developing techniques to approximate gradients of spike functions. Enhancements such as the iterative leaky integrate-and-fire (LIF) model (Wu et al., 2019) and the tdBN algorithm (Zheng et al., 2021) further improve training scalability and efficiency.

In our work, we leverage surrogate gradients due to their robustness in SNN training, which offers several advantages:

- **Temporal precision**: enhanced performance on time-sensitive tasks.
- **Architectural flexibility**: greater design freedom without the constraints of ANN architectures.
- **Energy efficiency**: optimized spike-based communication suitable for neuromorphic hardware.

While direct training may require more resources and time compared to conversion methods, its capacity to fully exploit SNN capabilities makes it a compelling choice for advancing performance.

### 2.3 Vision transformer in SNNs

ViT has significantly advanced image classification through self-attention mechanisms, capturing global dependencies and complex feature representations (Dosovitskiy et al., 2021). Typically, they consist of a patch splitting module, transformer encoder layers, and a classification head, with self-attention as a core component.

The integration of self-attention in SNNs represents an emerging area of research. Initial efforts have adapted ANN-Transformers for spike data; however, challenges remain in fully aligning these methodologies with the unique characteristics of SNNs (Yao et al., 2021; Zhang et al., 2022; Mueller et al., 2021).

Recent works, such as Spikformer and Meta-SpikeFormer explore the synergies between Transformers and SNNs (Zhou et al., 2023; Yao et al., 2024). These architectures aim to leverage spike-based self-attention to enhance energy efficiency and performance across various vision tasks. Notably, Meta-SpikeFormer has been evaluated on datasets like ImageNet-1K, demonstrating competitive accuracy and suggesting potential applications in neuromorphic computing.

Our research builds on these foundational works by further investigating self-attention mechanisms within SNNs, developing SpikeAtConv model to enhance computational efficiency and performance in image classification tasks.

## 3 Materials and methods

### 3.1 Overall architecture

In this study, we propose a spiking neural network architecture called SpikeAtConv, which is inspired by MaxViT (Tan and Le, 2019; Woo et al., 2023; Tu et al., 2022; Dai et al., 2021). The overall structure of the SpikeAtConv network is shown in Figure 1. MaxViT is a vision neural network architecture that effectively combines the strengths of Transformers and CNNs by integrating self-attention mechanisms with convolutional operations. Building on MaxViT, we modified both the Transformer and convolutional components to handle and generate spike signals, resulting in a novel spiking neural network model.

Firstly, the Feature Extraction Layer of the model consists of two convolutional layers and a SPK Block. Further details regarding the SPK Block will be provided in the following sections. The primary function of this layer is to downsample the input image, halving its resolution with a stride setting of 2 in the first convolutional layer. Additionally, it converts continuous image data into neural spike signals, i.e., binary discrete data, making it suitable for subsequent processing.

Next is the Feature Encoding Layer of the model, which forms the core of the model. It includes four stages, each performing downsampling at the entrance to halve the resolution of the feature map, with no further downsampling within the same stage. Each stage consists of a series of SpikeAtConv Blocks, varying in number but collectively achieving deep feature encoding. The SpikeAtConv Blocks represent our novel integration of CNN, attention mechanisms, and SNN, designed to enhance the performance of model. Detailed information about these modules is provided in subsequent sections. The depth of these four stages follows a spindle-shaped distribution; for instance, in the base model, the depths of the stages are 2, 6, 12, and 2, respectively. This design follows empirical rules of classification visual neural networks to effectively capture features and facilitate information flow.

Finally, the Decision Layer of the model is responsible for the classification task. It processes the output feature maps from the previous stages through global pooling, followed by a linear layer to predict the categories. This layer is designed to be both simple and efficient, capable of transforming complex, high-dimensional features into the final classification decision. The overall structure of our model leverages traditional methods while incorporating innovative SNN elements for enhanced performance.

### 3.2 SPK block

The LIF neuron model (Abbott, 1999; Gerstner et al., 2014) is a fundamental computational neuroscience model, widely used for its simplicity and reasonable approximation of biological neuron behavior. The core of the LIF model lies in simulating the dynamics of the neuronal membrane potential, which is governed by the following differential equation:


(1) $$\begin{array}{l} \tau \frac{dV}{dt}=-\left(V-V_{\text{rest}}\right)+RI\left(t\right) \end{array}$$

where *V* represents the membrane potential, τ is the membrane time constant, *V*_rest_ is the resting membrane potential, *R* is the membrane resistance, and *I*(*t*) is the input current. The resting potential *V*_rest_ is the membrane potential value of the neuron when it is in a resting state without any external input. If the neuron receives input from other neurons, the potential *V* will be deflected from its resting value. When the membrane potential *V* exceeds the threshold *V*_th_, the neuron fires a spike, and the membrane potential is reset to a lower reset potential *V*_reset_. In formal models of spiking neurons, the negative overshoot (spike-afterpotential) after the pulse is replaced by a “reset” of the membrane potential to the value *V*_reset_ (Gerstner et al., 2014). Subsequently, the neuron enters a refractory period during which it is unresponsive to new inputs. Other important hyperparameters in the LIF model include the duration of the refractory period, which affects the firing frequency and the response to consecutive inputs.

**Figure 2:**
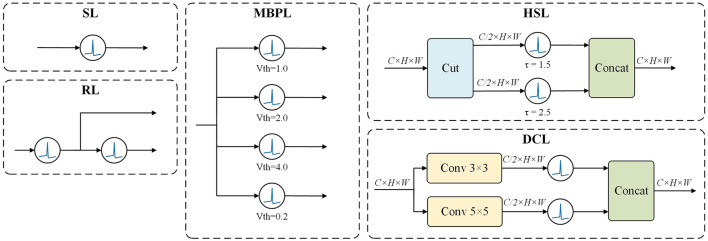
SPKBlock. Leveraging LIF neurons, we developed multiple SPK blocks to investigate the effects of various hyperparameters and different combinations of neurons on network performance. For instance, the MBPL Block features multiple parallel neurons with distinct thresholds, while the DCL Block comprises two parallel branches, each containing a convolutional layer followed by a LIF neuron.

Leveraging the dynamic properties of LIF neurons, we have designed five SPK Blocks, as illustrated in Figure 2, to emulate various aspects of biological neural network information processing mechanisms.

#### 3.2.1 Single-layer LIF SPK (SL) Block

This fundamental building block consists of a single LIF neuron. Despite its simplicity, it effectively simulates the activation and inhibition dynamics of an individual neuron.


(2) $$\begin{array}{l} s={\text{LIF}}_{1}\left(x\right) \end{array}$$

where *x* is the input signal, LIF_*i*_ denotes the LIF neuron.

#### 3.2.2 Residual LIF SPK (RL) Block

In this design, features pass through a LIF neuron and then split into two branches. The main branch is processed by a second LIF neuron, while the auxiliary branch retains the original features. The residual connection mitigates information loss and enhances the learning capacity of the model. Mathematically, this can be expressed as:


(3) $$\begin{array}{l} s={\text{LIF}}_{1}(x)\\ s=s+{\text{LIF}}_{2}(x)) \end{array}$$

where Concat represents the concatenation operation.

#### 3.2.3 Multi-branch parallel LIF SPK (MBPL) Block

This block comprises several LIF neurons with distinct hyperparameters arranged in parallel, allowing features to pass through several different thresholds simultaneously. The outputs of these neurons are then combined and fed into a ConvNeXt module to simulate membrane potential variations before summing the results. This approach enables the model to integrate information across different scales effectively. The formula is given by:


(4) $$\begin{array}{l} s=\text{Concat}\left({\text{LIF}}_{1,V_{\text{th}1}}\left(x\right),{\text{LIF}}_{2,V_{\text{th}2}}\left(x\right),\ldots ,{\text{LIF}}_{n,V_{\text{th}n}}\left(x\right)\right) \end{array}$$

where *V*_th*i*_ is the threshold voltage for the *i*-th LIF neuron.

#### 3.2.4 Hidden split LIF SPK (HSL) Block

This design splits and concatenates the outputs of two neurons along the hidden dimension. This method allows the model to capture features across different representational space dimensions, enhancing the model's expressive power. It is represented as:


(5) $$\begin{array}{l} x_{1},x_{2}=\text{Cut\_channel}\left(x\right)\\ \;s=\text{Concat}\left({\text{LIF}}_{1,\tau_{1}}\left(x_{1}\right),{\text{LIF}}_{2,\tau_{2}}\left(x_{2}\right)\right) \end{array}$$

where Cut_channel splits the input into separate channels, τ_*i*_ is the membrane time constant for the *i*-th LIF neuron.

#### 3.2.5 Dual convolutional LIF SPK (DCL) Block

The DCL Block features a bifurcated architecture with two parallel branches, each comprising a convolutional layer and a LIF neuron. The first branch harnesses a 3x3 convolutional kernel to discern fine spatial details, whereas the second branch leverages a 5x5 kernel to apprehend a wider spatial context. This strategy of extracting features at varying scales enables the DCL Block to simultaneously process spatial details with high and low resolution. Each branch's convolutional layer halves the channel dimension, and the outputs from both branches are subsequently concatenated along the channel axis, maintaining the original dimensionality. The mathematical representation is:


(6) $$\begin{array}{l} x_{1},x_{2}=\text{Cut\_channel}\left(x\right)\\ \;s=\text{Concat}\left({\text{LIF}}_{1}\left({\text{Conv}}_{3\times 3}\left(x_{1}\right)\right),{\text{LIF}}_{2}\left({\text{Conv}}_{5\times 5}\left(x_{2}\right)\right)\right) \end{array}$$

where Conv_3 × 3_ and Conv_5 × 5_ are convolutional filters of size 3x3 and 5x5, respectively.

### 3.3 Attention SpikeMerge Block

ViT was a pioneering effort to apply a pure Transformer architecture to image recognition, demonstrating the impressive capabilities of Transformers in image processing. However, ViT also revealed several challenges, such as optimization difficulties, convergence issues, and high computational and memory costs. Additionally, handling long-tail effects, intra-class variations, and designing effective positional encodings remain areas requiring further investigation.

MaxViT addresses these issues by incorporating the multi-axis self-attention (Max-SA) module, which balances local and global attention. The Max-SA module combines window attention with grid attention, providing a better inductive bias, and uses CNNs for positional encoding, thereby mitigating some of ViT's limitations.

**Figure 3:**
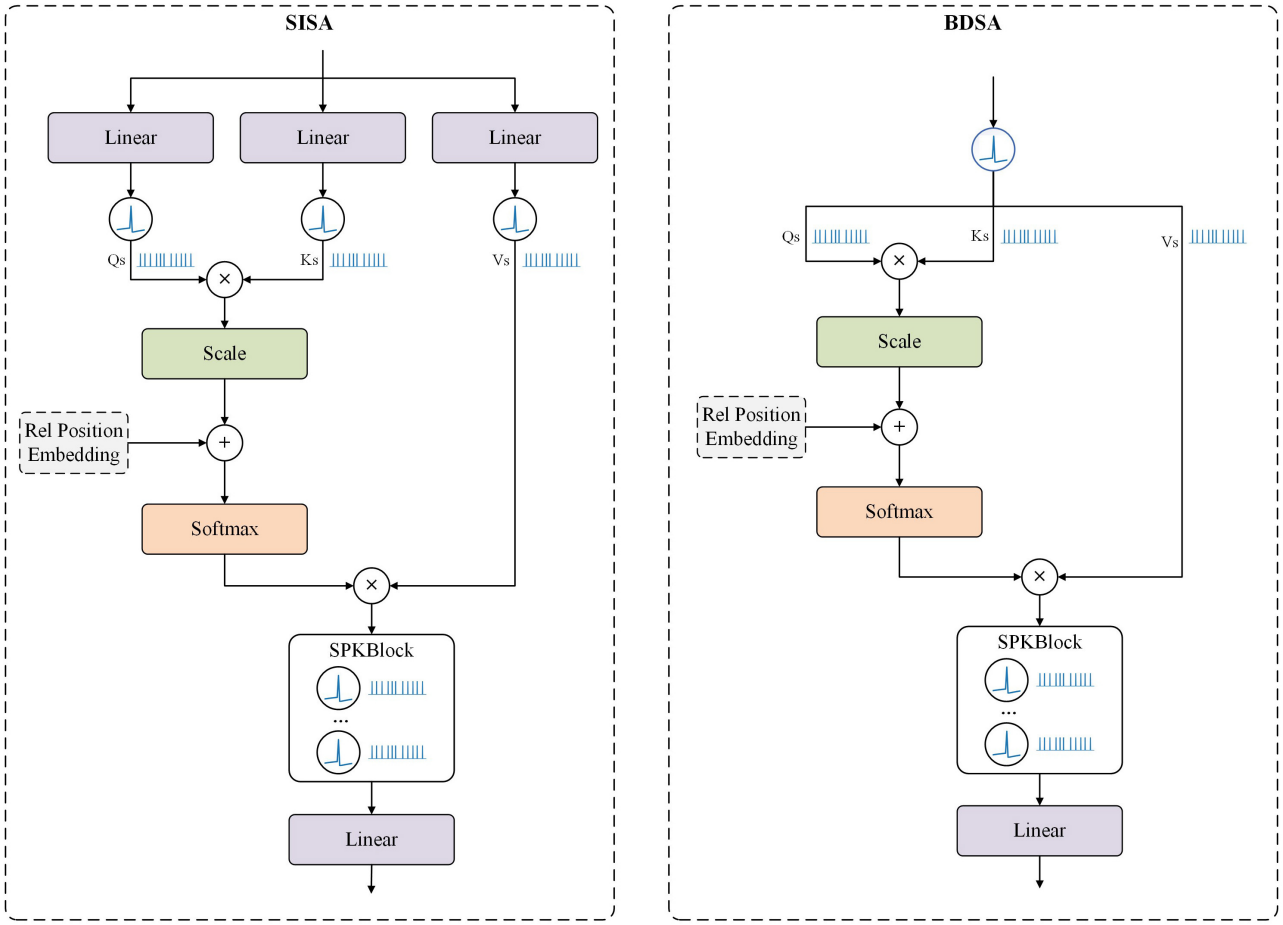
Attention SpikeMerge Block. This represents two different computational approaches. In the SISA Block, after computing the Q, K, and V, we add the SPK Blcok separately to obtain the spike form of Q, K, and V. Subsequently, we use Q and K to calculate the attention scores, apply these scores to V, and then incorporate the SPK Block to convert the attention into spike sequences. In the BDSA Block, we bypass the computation of Q, K, and V, directly converting the input into spike sequences through the SPK Block, treating Q, K, and V as the same.

Building on the MaxViT architecture, we propose two distinct Attention SpikeMerge Blocks that integrate the SPK module to process spike signals, as shown in Figure 3. Our goal is to optimize the combination of attention mechanisms with SPK modules based on LIF neurons to enhance spike signal processing.

In our implementation, we utilize multi-head self-attention as show in Figure 1, which is a standard approach in Transformer models. For clarity, we will primarily discuss self-attention in this section, as multi-head attention can be understood by simply adding the head dimension on top of the self-attention mechanism. This allows us to focus on the core principles while ensuring comprehensive coverage of our methods.

#### 3.3.1 Spike-integrated self-attention (SISA) block

In this approach, the SPK Block is incorporated during the computation of the self-attention query (Q), key (K), and value (V). After calculating the attention scores and applying them to V, the SPK Block converts the attention map into spike signals. In the Feed-Forward Network, each linear layer is followed by an Spk module to maintain the spike-based processing.

Mathematically, the SISA Block can be represented as:


(7) $$\begin{array}{l} \;Q_{S}=\text{LIF}\left(\text{Linear}\left(X\right)\right)\\ \;K_{S}=\text{LIF}\left(\text{Linear}\left(X\right)\right)\\ \;V_{S}=\text{LIF}\left(\text{Linear}\left(X\right)\right)\\ \text{Attention}=\text{Softmax}\left(\frac{Q_{S}K_{S}^{T}}{\sqrt{d_{k}}}+\text{Position Embedding}\right)V_{S}\\ \;\text{Output}=\text{SPKBlock}\left(\text{Attention}\right) \end{array}$$

where *X* is the input, and *d*_*k*_ is the dimension of the key vectors.

#### 3.3.2 Binary direct-spike attention (BDSA) Block

This approach diverges significantly from the previous one. Given the binary nature of spike signals, we bypass the computation of Q, K, and V and directly transform the input features into spike signals, treating Q, K, and V as identical. This method accelerates the computation of the attention map by using matrix multiplication with identical binary vectors. During inference, post-training, the attention-processed features can be directly obtained without additional computation, simplifying the process.

The BDSA Block can be described as:


(8) $$\begin{array}{l} \;Q_{S}=K_{S}=V_{S}=\text{LIF}\left(X\right)\\ \;\text{Attention}=\text{Softmax}\left(\frac{Q_{S}K_{S}^{T}}{\sqrt{d_{k}}}+\text{Position Embedding}\right)V_{S}\\ \text{Output}=\text{SPKBlock}\left(\text{Attention}\right) \end{array}$$

We trained models using these two distinct Attention SpikeMerge Blocks to evaluate the impact of varying module complexity on model performance. The experimental results and their implications will be discussed in the following sections.

## 4 Results

### 4.1 CIFAR-100 experiments

In this study, we utilized the CIFAR-100 dataset as a preliminary benchmark to evaluate and refine the design of various SPKBlock modules. CIFAR-100 is a well-regarded image classification dataset comprising 60,000 images across 100 distinct categories (Krizhevsky et al., 2009). Its manageable scale and diversity make it an ideal choice for initial experimentation aimed at optimizing computational resources and reducing experimental time.

To highlight the impact of SPKBlock modules, we deliberately refrained from optimizing the training parameters, instead opting for a straightforward and commonly used set of settings. Specifically, training was conducted over 200 epochs, AdamW optimizer with an initial learning rate of 0.02 (Loshchilov and Hutter, 2019). We opted for a batch size of 256 to ensure efficient use of computational resources while maintaining reasonable memory consumption. To mitigate early training instability, a warm-up strategy was implemented during the first 5 epochs. The weight decay parameter was set at 0.01 to counteract overfitting. Our primary goal was not to achieve a highly optimized model on CIFAR-100, but rather to evaluate the effectiveness of different SPKBlock modules.

In terms of data augmentation, we applied horizontal flipping, random rotations within a 30-degree range, and random shearing to enhance the model's generalization capabilities. Our base model architecture was ResNet-18, with various SPKBlock modules replacing the traditional activation functions to explore their impact. We systematically adjusted hyperparameters such as surrogate functions, voltage thresholds, tau values, and the spatial and temporal configurations of LIF neurons.

### 4.2 ImageNet1K experiments

In this study, we utilized the ImageNet-1K dataset as a benchmark to evaluate and compare the efficacy of different neural network module designs (Deng et al., 2009). ImageNet-1K is a widely-used image classification dataset that contains over one million annotated images across one thousand distinct categories. Its diversity and scale make it a significant challenge in the field of computer vision.

Regarding our experimental setup, we employed a series of meticulously chosen training parameters. Specifically, we set our training to run for 200 epochs to ensure ample learning opportunities. The initial learning rate was set at 0.001, a value aimed at balancing convergence speed and training stability. We opted for a batch size of 768 to make efficient use of our computational resources while maintaining reasonable memory consumption. During the first 10 epochs, we implemented a warm-up strategy to mitigate early training instability. The weight decay parameter was set at 0.05 to help counteract overfitting. The gradient clipping threshold was established at 0.1 to prevent gradient explosion issues. All images were resized to a uniform resolution of 224 × 224 to maintain consistency in input data.

Additionally, we adopted a cosine learning rate decay strategy, which allows for a smooth reduction of the learning rate in the later stages of training, aiding the model in converging to a more optimal solution. For data augmentation, we utilized the AutoAugment technique, an approach that optimizes augmentation policies through automatic searching. We also employed label smoothing with a value of 0.1 to reduce the model's sensitivity to label noise. Techniques such as Random Erase, Mixup, and CutMix were integrated as well, which have been proven to effectively enhance the model's generalization capabilities on images (Zhang and Deng, 2021; Yun et al., 2019).

We designed three different model architectures to explore the impact of varying network scales on performance: Tiny, Base, and Large models.

For the **Tiny** model, the hidden state dimensions were set to 64, 128, 256, and 512 for the four stages, respectively. The module depths for each stage were set at 1, 3, 6, and 1. This configuration aims to provide a lightweight model suitable for environments with limited computational resources.

For the **Base** model, the hidden state dimensions were set to 128, 256, 512, and 1024 for the four stages, respectively. The module depths for each stage were set at 2, 6, 12, and 2. This design is intended to progressively extract and process features of the images, balancing computational efficiency and performance.

For the **Large** model, the hidden state dimensions were set to 160, 320, 640, and 1280 for the four stages, respectively. The module depths for each stage were set at 2, 6, 16, and 2. This configuration aims to capture more complex features and provide higher accuracy, suitable for environments where computational resources are abundant.

#### 4.2.1 Theoretical energy consumption evaluation

In this work, we utilize a theoretical framework to assess the energy consumption of SNNs with the goal of contrasting them against conventional ANNs. Our methodology draws upon the approaches detailed in Li et al. (2021b) and Yao et al. (2024). Furthermore, we have evaluated the energy consumption of various models based on experiments performed on the ImageNet1K dataset. The energy consumption assessment hinges on several critical parameters and equations.

Initially, we establish two key energy consumption metrics:

**Energy cost per multiply-accumulate operation** (***E**_*MAC*_*): For the purposes of this study, we estimate *E*_*MAC*_ to be 4.6 pJ, which represents the energy expended for executing one multiplication followed by one addition.

**Energy cost per addition operation** (***E**_*A*_*): We assume *E*_*A*_ to be 0.9 pJ, denoting the energy required for a single addition operation.

Subsequently, we compute the Floating Point Operations per Second (FLOPs) across various layers, which is a vital factor in determining the energy consumption of neural networks. For convolutional layers (Conv), the FLOPs can be calculated using the formula:


$${\text{FLOPs}}_{\text{Conv}}={\left(k_{n}\right)}^{2}\cdot h_{n}\cdot w_{n}\cdot c_{in}\cdot c_{out}$$

where *k*_*n*_ signifies the size of the convolutional kernel, *h*_*n*_ and *w*_*n*_ represent the height and width of the resulting feature map, while *c*_*in*_ and *c*_*out*_ denote the number of input and output channels, respectively. For Multi-Layer Perceptrons (MLPs), the FLOPs calculation is expressed as:


$${\text{FLOPs}}_{\text{MLP}}=n_{in}\cdot n_{out}$$

where *n*_*in*_ and *n*_*out*_ are the dimensions of the input and output layers of the MLP.

Moreover, the Spiking Rate (R) is defined as the ratio of non-zero elements present in the spike tensor, which reflects the sparsity of the neural network during its operation. In our calculations, the average spiking rate is derived from the spiking rates of various spike tensors.

Finally, the overall energy consumption for the Spiking Neural Network is computed using the following equation:


$$\begin{array}{l} \text{Total Energy Consumption}=E_{A}\times T\times R+E_{MAC}\\ \times \text{(Total Multiply-Accumulate Operations)} \end{array}$$

where *T* represents the time step, and *R* is the spiking rate. We posit that under specific conditions (i.e., *E*_*A*_×*T*×*R*<*E*_*MAC*_), Spiking Neural Networks demonstrate a favorable energy consumption profile.

### 4.3 Main properties

We ablate SpikeAtConv using the default settings from Section 4.1 and observe some interesting phenomena.

#### 4.3.1 Comparative analysis of SPK block variants

To evaluate the performance of different SPK blocks, we selected SISA as the Attention SpikeMerge Block. Our experiments on the CIFAR-100 dataset systematically assessed various SPKBlock configurations, focusing on the impact of different simulation time windows, surrogate gradient functions, and the number of branches on model accuracy. Table 1 presents a detailed comparison of these configurations.

**Table 1:** Accuracy for different SPKBlock on CIFAR-100.

**Model**	**Top-1 Acc (%)**	**SPKBlock**	***T***	**τ**	**V Threshold**	**Number of branches**	**Surrogate**
ResNet-18	75.4	—	—	—	—	—	—
ResNet-18	64.3	SL	1	4.0	1.0	1	Sigmoid
ResNet-18	66.0	SL	1	2.0	1.0	1	Sigmoid
ResNet-18	67.1	SL	1	2.0	1.0	1	ATan
ResNet-18	70.9	SL	4	2.0	1.0	1	Sigmoid
ResNet-18	70.4	SL	8	2.0	1.0	1	Sigmoid
ResNet-18	71.0	RL	1	2.0	1,2	2	ATan
ResNet-18	74.4	MBPL	2	2.0	0.2,1,2,4	4	ATan
ResNet-18	70.6	HSL	2	2.0	1,2	2	ATan
ResNet-18	70.3	DCL	2	2.0	1,1	2	ATan

Surrogate denotes surrogate gradient, a smooth approximation used to train spiking neural networks.

The analysis of SL Block configurations revealed that increasing the simulation time window *T* generally enhances model performance. For instance, the accuracy improved from 66.0% at *T* = 1 to 70.9% at *T* = 4. However, when *T* was further increased to *T* = 8, there was a slight performance drop to 70.4%. This indicates an optimal range for *T*, beyond which the benefits diminish, likely due to increased complexity and potential overfitting.

Additionally, the choice of surrogate gradient functions significantly impacted performance. The Atan function outperformed the Sigmoid function under equivalent settings. For example, with *T* = 1 and τ = 2.0, the accuracy with Atan was 67.1%, compared to 66.0% with Sigmoid. This suggests that the Atan function provides a more effective gradient approximation for training spiking neurons under these specific conditions.

In the MBPL Block experiments (see Table 2), we observed that increasing the number of branches markedly enhanced performance. A configuration with four branches and varied voltage thresholds achieved a top-1 accuracy of 74.4%, significantly higher than simpler configurations. However, an excessive number of branches, such as eight, resulted in a performance drop to 59.0%. This decline was attributed to the insufficient training of the numerous branches, which introduced noise and hampered the model's learning capacity.

**Table 2:** MBPL Block experiments on CIFAR-100.

**Model**	**Top-1 Acc (%)**	**SPKBlock**	***T***	**τ**	**V Threshold**	**Number of branches**	**Surrogate**
ResNet-18	71.7	MBPL	1	2.0	1,2	2	ATan
ResNet-18	71.9	MBPL	1	2.0	1,2,4	3	ATan
ResNet-18	72.5	MBPL	1	2.0	0.2,1,2,4	4	ATan
ResNet-18	72.2	MBPL	1	2.0	1,2,4,6	4	ATan
ResNet-18	73.8	MBPL	2	2.0	0.2,1,2,4	4	Sigmoid
ResNet-18	74.4	MBPL	2	2.0	0.2,1,2,4	4	ATan
ResNet-18	73.6	MBPL	4	2.0	0.2,1,2,4	4	ATan
ResNet-18	59.0	MBPL	2	2.0	0.2,1,2,3,4,5,6,7	8	ATan

In our CIFAR-100 experiments, we only listed representative examples. For the HSL Block, when using the same number of LIF branches, its performance was similar to that of the MBPL Block. However, it is either computationally more complex or less scalable, so we did not list more detailed results.

Through extensive experimentation, we discovered that the MBPL module exhibited the best overall performance. For each module, we identified the optimal hyperparameter settings and structural configurations specific to CIFAR-100. These preliminary results allowed us to eliminate numerous suboptimal designs and provided valuable insights for further experiments on more complex datasets like ImageNet-1K.

Table 3 provides a detailed performance analysis of various SPKBlock configurations combined with Attention SpikeMerge Blocks on the ImageNet-1K dataset. The results indicate that the MBPL modules consistently outperform other configurations, demonstrating superior accuracy and scalability. This establishes MBPL as our default choice due to its ability to effectively integrate information across multiple LIF neurons, thereby compensating for potential deficiencies in individual neuron processing capabilities.

**Table 3:** Accuracy for different SPKBlock and attention SpikeMerge Block on ImageNet1K.

	**Top1 (%)**	**Top5 (%)**
SL+SISA	74.91	91.94
RL+SISA	78.35	93.73
MBPL+SISA	80.53	94.17
HSL+SISA	78.44	93.75
DCL+SISA	77.66	93.83
MBPL+BDSA	77.63	93.63
spike-free	81.13	94.30

Additionally, it is noted that the RL module outperforms the SL module in terms of performance. This improvement can be attributed to the use of a residual structure, where features are split into two branches after passing through the first LIF neuron. The main branch is further processed by the second LIF neuron, while the auxiliary branch retains the original features. This design effectively mitigates information loss and enhances the learning capacity by preserving and refining feature representations.

However, despite the theoretical advantages, we found that DCL modules prevented the model from being fully trained. After 40 epochs, the loss of the training set stopped decreasing. Even lowering the learning rate, adjusting the position of the SPK module, or adding a normalization layer did not resolve this issue. This is a common problem encountered when processing spiking signals, where the neural spike module is prone to crashing in the existing deep learning training framework.

Following the selection of MBPL, we further examined the impact of different Attention SpikeMerge Blocks. Notably, the BDSA structure, characterized by its simplicity, allows for a significant simplification of the attention computation. By treating the Q, K, and V matrices as identical binary matrices composed of 0s and 1s, the matrix multiplication process is streamlined. Although this simplification led us to anticipate a reduction in performance compared to the more complex SISA structure, the results were surprising. The MBPL+BDSA configuration exhibited only a marginal decrease in performance, suggesting that even with a simplified attention mechanism, the model maintains a robust level of effectiveness. This finding underscores the potential of BDSA to offer computational efficiency without substantial loss in accuracy, paving the way for further algorithmic optimizations.

#### 4.3.2 Comparison of SpikeAtConv and other models on ImageNet-1K

We evaluated the performance of our SpikeAtConv model at different scales (Tiny, Base, and Large) on the ImageNet-1K dataset. Each model was trained for 200 epochs with an image input resolution of 224. Our findings indicate that while the Large model achieves slightly higher accuracy than the Base model, further improvements are anticipated with increased image resolution and additional training epochs.

Table 4 presents a detailed comparison of the performance metrics, including top-1 and top-5 accuracy, the number of parameters, and power consumption for each model. The SpikeAtConv models are also compared against state-of-the-art models like Meta-SpikeFormer and SpikFormer.

**Table 4:** Performance of different models on ImageNet-1K.

**Model**	**T**	**Top-1 (%)**	**Top-5 (%)**	**Param (M)**	**Power (mJ)**
RMP-SNN-VGG-16 (Han et al., 2020)	40.96	73.09	—	—	—
Dspike-VGG-16 (Li et al., 2021b)	5	71.24	—	—	—
Spike-driven Transformer (Yao et al., 2023)	4	77.07	—	66.3	6.1
SpikFormer (Zhou et al., 2023)	4	74.81	—	66.3	21.5
Meta-SpikeFormer (Yao et al., 2024)	1	79.1	—	55.4	13.0
Base	1	80.53	94.17	53.4	14.2
Base	2	80.70	94.89	53.4	28.2
Tiny	1	76.58	92.74	14.6	3.8
Large	1	81.23	95.41	116.1	32.0

From the table, it is evident that our Large SpikeAtConv model achieves a top-1 accuracy of 81.23%, outperforming both Meta-SpikeFormer (79.1%) and SpikFormer (74.8%). The Base model also shows competitive performance with a top-1 accuracy of 80.53%. Despite having fewer parameters, the Tiny model maintains a respectable top-1 accuracy of 76.58%, demonstrating the efficiency of our approach.

In terms of power consumption, the Large model consumes 32.0 mJ, which, while higher than the Base model's 14.2 mJ, is still competitive when compared to traditional models like RMP-SNN-VGG-16 (64.9 mJ) and Dspike-VGG-16 (80.3 mJ). The Tiny model stands out with the lowest power consumption of 3.8 mJ, further highlighting the efficiency of smaller models without a significant sacrifice in accuracy.

The comparison indicates that SpikeAtConv models, particularly the Large variant, provide superior performance on ImageNet-1K while maintaining a balance between accuracy, model complexity, and energy efficiency. This demonstrates the effectiveness of our approach in leveraging SNNs for large-scale image classification tasks, making them a promising choice for applications where both performance and energy consumption are critical factors.

## 5 Discussion

In summary, our results highlight two key insights. First, a well-designed SNN architecture can significantly enhance the performance of SNNs. Second, integrating SNNs with advanced deep learning architectures can further improve their effectiveness.

For the first point, we observed that SNN modules based on LIF neurons tend to lose a considerable amount of information. This refers not only to information relevant to classification tasks but also to high-level semantic information present in images, such as color, contours, and other detailed features. While such information might be redundant for specific classification outcomes, it is crucial for a comprehensive understanding of the input data. The SNN module functions similarly to an activation function, filtering out these details. However, this filtering can inadvertently lead to performance degradation by omitting potentially valuable contextual information. To address this, we adopted an approach akin to early CNNs by setting up parallel LIF neurons with different parameters. This setup captures varying levels of information, thereby maximizing the richness of information extracted by the SNN.

**Figure 4:**
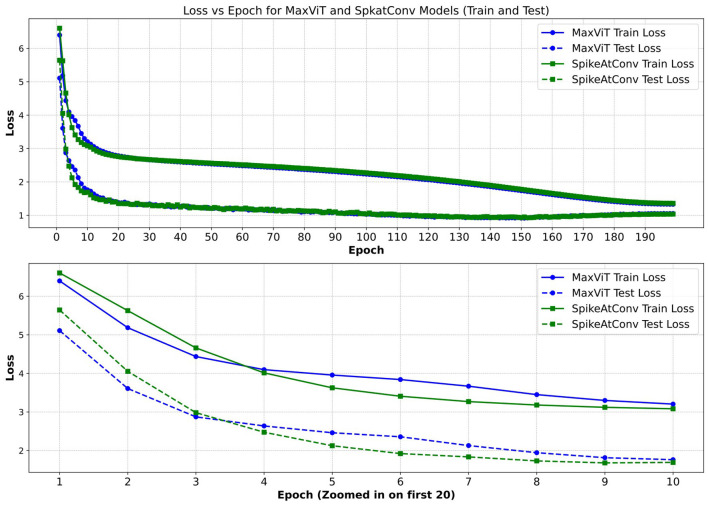
Comparison of loss between MaxViT and SpikeAtConv. We present the training and validation loss trajectories for our SpikeAtConv and MaxViT models, with a particular emphasis on the loss variations during the first 10 epochs. Notably, SpikeAtConv exhibits a slower decrease in loss during the initial five epochs. Additionally, the application of data augmentation techniques, such as auto-augmentation and mixup, results in the training loss consistently remaining higher than the test loss.

Regarding the second point, our experiments demonstrate that the reasonable integration of SNN modules can have minimal impact on the original performance of the neural network. However, we also noticed that the loss of the SNN network decreased more slowly in the early stages of training compared to networks without SNNs (Figure 4). This suggests that while SNNs have the potential to achieve excellent results in visual tasks, further research is needed to develop training methods and network modules that effectively cooperate with SNNs.

Traditional deep learning architectures like CNNs and Transformers have been extensively researched, leading to the development of numerous auxiliary layers, targeted data augmentation techniques, and pre-training strategies that ensure these networks are well-trained and stable. Similarly, SNNs require further in-depth studies to develop analogous methods that can ensure sufficient training and stability during the training process. This includes designing specialized layers, data augmentation techniques, and training protocols tailored specifically for SNNs to unlock their full potential.

In addition, we designed the BDSA module based on the hypothesis that the complexity of traditional self-attention mechanisms might not yield significant benefits when processing simple spiking signals. Consequently, BDSA simplifies the attention block considerably. Surprisingly, the results exceeded our expectations, as the BDSA module did not result in a substantial performance drop. This suggests that for spiking signals, simpler and more suitable attention blocks can be explored. Such exploration could not only reduce training and inference costs but also deepen our understanding of spiking signals and the reasoning mechanisms of the human brain. This line of inquiry opens up new avenues for developing efficient and interpretable SNN architectures.

## 6 Conclusion

In this study, we developed a novel spiking neural network model named SpikeAtConv, which achieved SOTA results among SNN models on the ImageNet-1K dataset. Our approach involved designing a series of SPK blocks to convert continuous hidden states into neural spikes. Through extensive experimentation, we identified the optimal SPK block configuration and integrated it with the MaxVit architecture. This combination enabled us to significantly advance the performance of SNNs.

One of our key findings was that even when using a degenerate self-attention mechanism, the performance of our model did not degrade significantly. This suggests that our SPK blocks are highly effective in capturing and processing information, even without the full complexity of self-attention.

Additionally, our experiments demonstrated that a well-designed SNN architecture can substantially enhance performance. By setting up parallel LIF neurons with different parameters, we were able to capture various levels of information, thereby enriching the data representation within the SNN.

Looking forward, we aim to further refine the design of SPK blocks and explore improvements in backpropagation techniques. These enhancements will help ensure that SPK blocks are fully trained and can further improve the performance and stability of SNNs across various tasks. Moreover, we recognize the need for developing specialized training methods, auxiliary layers, and data augmentation techniques tailored specifically for SNNs, akin to the extensive research conducted for CNNs and Transformers.

In conclusion, our work not only introduces a powerful new SNN model but also lays the groundwork for future research in optimizing SNN architectures and training methodologies. We believe that with continued exploration and innovation, SNNs can achieve even greater performance and applicability in diverse domains.

## Data Availability

The original contributions presented in the study are included in the article/supplementary material, further inquiries can be directed to the corresponding author.
